# Purification and identification of two novel antioxidant peptides from perilla (*Perilla frutescens* L. Britton) seed protein hydrolysates

**DOI:** 10.1371/journal.pone.0200021

**Published:** 2018-07-09

**Authors:** Juanjuan Yang, Lei Hu, Tiantian Cai, Qiuluan Chen, Qian Ma, Jie Yang, Chun Meng, Jing Hong

**Affiliations:** College of Biological Science and Technology, Fuzhou University, Fuzhou, Fujian, PR China; Institute of medical research and medicinal plant studies, CAMEROON

## Abstract

Proteins were extracted from perilla (*Perilla frutescens* L. Britton) seed by-products and hydrolyzed with an alkaline protease. Antioxidant peptides were purified from the hydrolysate by size-exclusion chromatography and RP-HPLC. Two peptides with strong antioxidant activity were identified as Tyr-Leu (YL) and Phe-Tyr (FY) with the molecular mass of 294.33 Da and 328.33 Da, respectively. Synthesized YL and FY efficiently quenched free radicals (DPPH, ABTS and hydroxyl radicals) and showed high oxygen radical absorbance capacity. The two peptides also inhibited lipid peroxidation in the rat liver. Furthermore, YL and FY could protect HepG-2 cells against hydrogen peroxide-induced oxidative damage without cytotoxicity. Based on the structure-activity analysis, the Tyr residue was crucial for the antioxidant activity of YL and FY. The results indicate that the protein hydrolysate from perilla seed by-products possessed potent biological activity and can be utilized to develop health-related nutraceutical ingredients.

## Introduction

During cell metabolism, reactive oxygen species (ROS) are produced. Excessive ROS can cause oxidative stress and damages to membranes, proteins, and DNA, leading to development of chronic disease [1]. Under normal circumstances, the organism has the ability to eliminate endogenous free radicals. Nevertheless, when metabolism is disordered or attacked by exogenous free radicals, the balance between endogenous antioxidants and free radicals could be broken. Synthetic chemical antioxidants such as butyl hydroxy anisole (BHA), butylated hydroxytoluene (BHT), and tert-Butylhydroquinone (TBHQ) have strong antioxidant activity and are commonly used in the food industry[2]. However, these antioxidants possess potential risks, thus their usage should be strictly limited[3]. Searching of safe, natural antioxidant alternatives to synthetic antioxidants has become a hot research area. Compared with chemical antioxidants, antioxidant peptides can offer potential health benefits with low molecular weight, high activity, easy absorption, little or no negative side effects, and fast clearance from the blood[4], which attracts growing research interest. For instance, peptides separated from hemp seeds[5], Chinese leek seeds[6] and Palmaria palmata proteins[7] exhibited strong antioxidant activity *in vitro*.

Perilla (*Perilla frutescens* L. Britton) is an annual plant in the family of *Lamiaceae*, and is widely cultivated in East Asian countries including China, Japan, South Korea, and India[8]. Perilla stem leaves and seeds are used in traditional Chinese herbal medicine[9]. Despite high protein contents, defatted perilla seed by-products is only used as animal feed or fertilizer[10]. Therefore, it is worthwhile that perilla seed by-products is utilized as a source of bioactive peptides. In this study, we purified and characterized antioxidant peptides from defatted perilla seed protein hydrolysate (PSPH). The protective effects of the antioxidant peptides against H_2_O_2_-induced oxidative damage in HepG-2 cells were studied. Furthermore, structure-activity relationship of the peptides was investigated.

## Materials and methods

### Materials

Perilla seeds were purchased from a local seeds market in Fuzhou, China. 1,1-diphenyl-2-picryl-hydrazil (DPPH) and 2,2’-azino-bis (3-ethylbenzothiazoline-6-sulphonic acid) diammoniumsalt (ABTS) were obtained from Sigma-Aldrich Co. (Pasadena, TX, USA). Acetonitrile, formic acid and trifluoroacetic acid (TFA) were of chromatographic grade and purchased from Sinopharm Chemical Reagent Co., Ltd (Fuzhou, China). Sephadex G-25 was purchased from GE Healthcare (Gothenburg, Sweden). RPMI-1640, fetal bovine serum (FBS), penicillin and streptomycin were purchased from Xiamen Lulong Biological Technology Development Co., Ltd (Xiamen, China). Other chemicals and solvents of analytical grade were obtained from different commercial sources. The identified peptides were synthesized by GL Biochem Ltd. (Shanghai, China), with purity higher than 98% by HPLC analysis.

### Preparation of antioxidant peptides

The defatted perilla seed meal was dissolved in NaOH solution (pH 9.4) at a ratio of 1:11 (w/v) and stirred at 58 °C for 1 h. The extract was centrifuged at 12, 000 rpm for 15 min at 4 °C, and the supernatant was adjusted to pH 4.0 with 1M HCl. The mixture was centrifuged at 12, 000 rpm for 15 min at 4 °C, and the resulting precipitate was washed and lyophilized. The freeze-dried proteins were mixed with distilled water at a ratio of 3% (w/v), and the pH of the mixture was adjusted to 9.8 with 2 M NaOH. Alkaline protease was added at a ratio of 3, 000 U/g and incubated at 50 °C for 5 h. After incubation, the reaction was terminated by heating at 100 °C for 10 min. The perilla seed protein hydrolysate (PSPH) was centrifuged at 10, 000 rpm for 10 min. The supernatant was collected, lyophilized and stored at -20 °C for further analysis.

### Purification of antioxidant peptides

The freeze-dried PSPH was dissolved in deionized water and filtered using a 0.22 μm membrane, and then loaded onto a Sephadex G-25 column (Φ1.6×100 cm). The peptides were eluted by deionized water at a flow rate of 0.3 mL/min. The elution curves were obtained by measuring absorbance at 214 nm using a spectrophotometer. All the peak fractions were collected and evaluated by DPPH radical scavenging activity. The fraction with the highest DPPH radical scavenging activity was filtered through a micro-filtration membrane (0.22 μm) and injected into a semi-preparative C-18 RP-HPLC column (Φ10 mm × 250 mm) on a Shimadzu LC-20A RP-HPLC system (Kyoto, Japan). The peptides were eluted with a linear gradient of acetonitrile (ACN) (5–40%) containing 0.05% TFA at a flow rate of 2.0 mL/min. All eluted fractions were detected at 214 nm, pooled and lyophilized for further study.

### Identification of peptides by LC-ESI-MS/MS

Molecular mass and sequences of the purified peptides were determined using a Q-TOF MS coupled with a Waters Acquity UPLC system through an electrospray ionization (ESI) source (Waters Corp., Milford, MA, USA). All experiments were carried out in the positive mode with a capillary voltage of 3, 500 V. Data were gathered in the centroid mode covering the mass/charge range from 0 to 1, 000 m/z, and the spectra were analyzed with the Masslynx software (Waters Corp.).

### Antioxidant activity of the synthetic peptides

#### DPPH and ABTS radical scavenging activity

DPPH and ABTS radical scavenging assays were determined following our previously published method [6]. Distilled water and reduced glutathione (GSH) replaced samples served as the blank and positive control, respectively. The scavenging effect was calculated according to the following equation:

$$\text{DPPH}/\text{ABTS}\;\text{radical}\;\text{scavenging}\;\text{activity}(\text{\%})\;=\;\frac{{\text{A}}_{\text{blank}}-{\text{A}}_{\text{sample}}}{{\text{A}}_{\text{blank}}}\times 100$$

#### Superoxide anion radical-scavenging activity

Superoxide anion radical-scavenging assay was performed with our published method reported [6]. Distilled water and GSH were included in the assay as the blank and positive control, respectively. The superoxide radical-scavenging activity was calculated as follows:

$$\text{Superoxide}\;\text{radical}\;\text{scavenging}\;\text{activity}(\%)\;=\;\frac{\Delta {\text{A}}_{0}/\text{min}-\Delta {\text{A}}_{\text{s}}/\text{min}}{\Delta {\text{A}}_{0}/\text{min}}*100$$

where ΔA_0_ /min is the slope of the absorbance line of blank control; ΔA_s_ /min is the slope of the absorbance line of the sample mixture.

#### Hydroxyl radical scavenging activity

The hydroxyl radical scavenging activity was assayed by using the method described by Y. Zhang *et al*. with some modifications[11]. The reaction mixture, containing 0.335 mL of sample solution, 0.1 mL of FeSO_4_ (8 mM), 0.335 mL of salicylic acid (3 mM) and 0.08 mL of H_2_O_2_ (20 mM) was incubated at 37 °C for 30 min. The reaction was cooled by flowing water to room temperature. Then 0.15 mL distilled water was added to the mixture to make the final volume 1.0 mL, and the mixture was centrifuged at 3000 ×g for 10 min. The absorbance of the supernatant was measured at 510 nm. Distilled water and GSH solution were used as blank and positive control, respectively. The capability of scavenging the hydroxyl radical was calculated as follows:

$$\text{Hydroxyl}\;\text{radical}\;\text{scavenging}\;\text{activity}(\%)\;=\;\frac{{\text{A}}_{\text{blank}}-{\text{A}}_{\text{sample}}}{{\text{A}}_{\text{blank}}}*100$$

#### ORAC assay

ORAC is another evaluation for antioxidants, which was measured according to previously published method[12]. Sodium phosphate buffer and GSH were used as the blank and positive control, respectively. ORAC values of peptide were calculated with the standard curve prepared with Trolox (0–100 μM). The area under the fluorescence decay curve (AUC) was calculated as follows:

$$\text{AUC}\;=\;\sum_{i\;=\;0}^{i\;=\;100}\frac{f_{i}}{f_{0}}$$

where *f*_0_ is the initial fluorescence reading at 0 min and *f*_i_ is the fluorescence reading at *i* min. The net-AUC was obtained by subtracting the AUC of the blank from that of each sample and demonstrated linear relationship with antioxidant activity of sample. The resulting ORAC values were expressed as μM trolox equivalent (TE)/ mg peptide.

### Inhibition of lipid peroxidation

The lipid peroxidation inhibition activity of antioxidant peptides was measured in a linoleic acid emulsion system according to previously published method [6]. Deionized water was used as the blank, and GSH and BHA were used as positive controls.

### Inhibition of lipid peroxidation in the rat liver

Kunming rats were purchased from Slac laboratory animal, China. Animals were treated in accordance with the Guide for the Care and Use of Laboratory Animals (Ministry of Science and Technology of China, 2006) and the study was approved by Fuzhou University Institutional Animal Care and Use Committee. The liver was collected from female rats (weighing 18–22 g, 8-week-old), washed with normal saline in ice bath three times, blotted dry and weighed. Nine volumes of normal saline were added to the beaker with rat liver, which was cut into pieces with ophthalmic scissors quickly. The mixture was homogenated in ice-bath with a homogenizer for 6–8 min. The homogenate was centrifuged at 4, 000 rpm for 10 min, and the supernatant was 10% (W/V) liver homogenate.

The capacity of the peptides to inhibit lipid peroxidation in the rat liver was tested using the method reported by Zeng *et al*. [13]. Distilled water and GSH were used as the blank and positive control, respectively. The inhibition of lipid peroxidation in rat liver was calculated as (1-A _sample 532_/A _blank532_) × 100%.

### Cell assays

#### Cell cultures

Human hepatoma G2 (HepG-2) cells (Cell resource center, Shanghai Institutes for Biological Science, Chinese Academy of Science, Shanghai, China) and Chinese Hamster Ovary (CHO) cells (GenScript, Nanjing, China) were cultured in RPMI-1640 containing 10% (v/v) fetal bovine serum, 100 U/mL penicillin and 100 μg/mL streptomycin in a humidified atmosphere with 5% CO_2_ at 37 °C. The culture medium was changed every other day. Cells were used for assays when they attained approximately 80% confluence.

#### Cytotoxicity assay

The cytotoxicity assay was carried out using the Annexin V-FITC and propidium iodine (PI) dual staining apoptosis detection kit (KeyGEN Biotech, China). Cells at a concentration of 2×10^5^ cells/well in 6-well plates with 2 mL culture medium per well were treated with the antioxidant peptides for 24 h. The cells were detached by pancreatin without EDTA and washed with cold phosphate buffered saline. Detached cells were resuspended in 500 μL binding buffer and incubated with 5 μL Annexin V-FITC and 5 μL PI in the dark for 10 min. The processed cells were immediately detected by flow cytometry to measure the cell apoptosis rate. The annexin V positive population (annexin V+/PI+ and annexin V+/PI-) was regarded as apoptotic cells. The annexin V negative and PI negative population (annexin V-/PI-) was regarded as normal cells.

#### Protective effects against H2O2-induced oxidative injuries in HepG-2 cells

HepG-2 cells were seeded in 6-well plates at a density of 2×10^5^ cells/well and grew in 2 mL growth media for 24 h. The cells were then incubated with antioxidant peptides for 18 h, followed by exposure to 1 mM H_2_O_2_ for another 6 h. Cell apoptosis rate was determined with Annexin V-FITC and PI dual staining as described above. In the H_2_O_2_-treated group, cells were treated with 1 mM H_2_O_2_ without peptides.

### Statistical analysis

All experiments were carried out with at least three independent replicates (n = 3), and all data were expressed as means ± standard deviation. Analysis of variance (ANOVA) was performed by SPSS 19.0 (SPSS, Chicago, IL, USA). The significance in differences was determined by Duncan’s multiple range test (*p* < 0.05).

## Results and discussion

### Purification of antioxidant peptides

**Fig 1:**
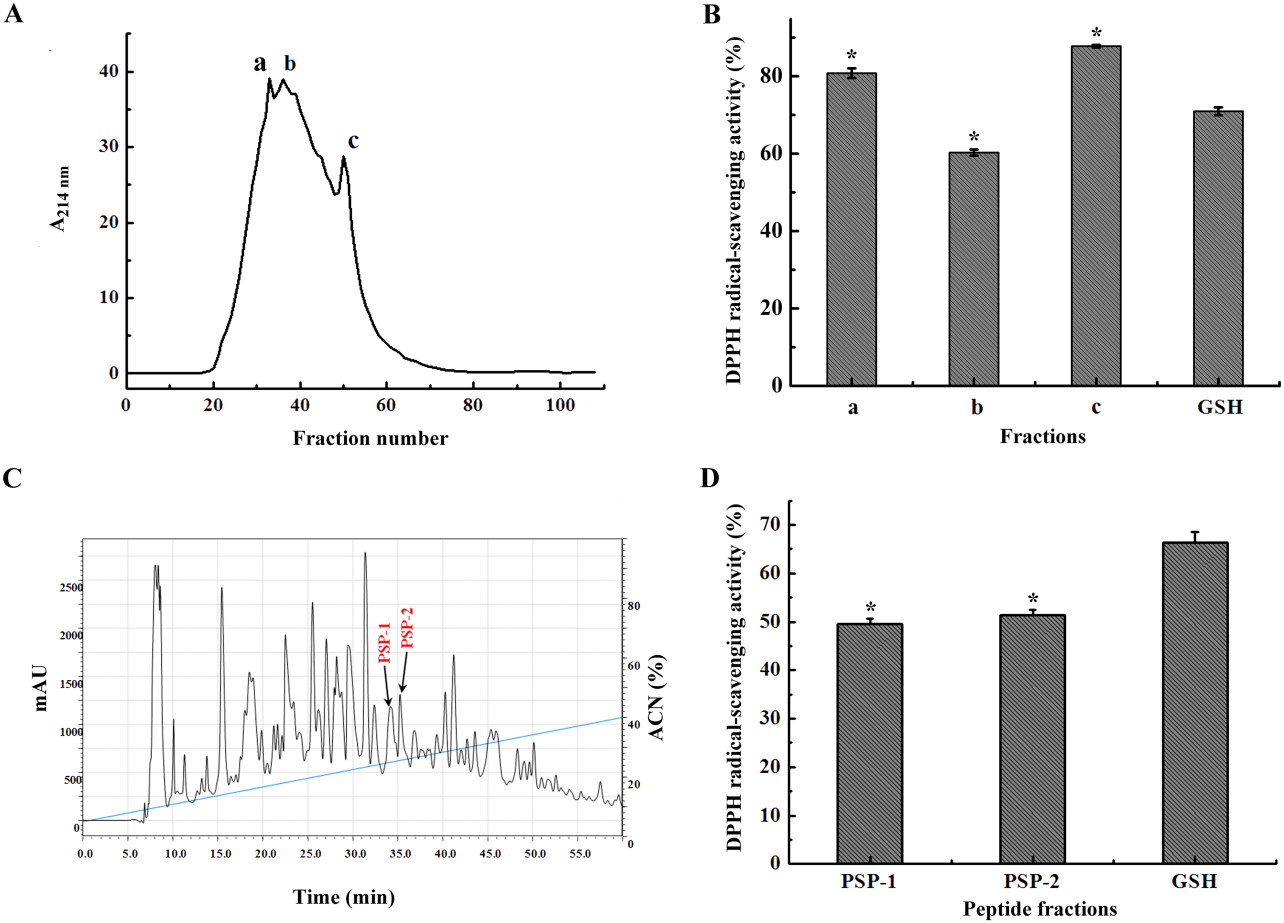
Purification of the perilla seed antioxidant peptides. A: Elution profile of perilla seed proteins enzymatic hydrolysates separated by gel filtration chromatography on a Sephadex G-25 column; B: DPPH radical-scavenging activity of the eluted fractions from the Sephadex G-25 column at the concentration of 0.5 mg/mL; C: RP-HPLC chromatogram of fraction c from Sephadex G-25 chromatography. The eluted fractions were detected at 214 nm. The gradient line shows the concentration of acetonitrile in the eluting solvent, and the eluted flow was 2.0 mL/min. D: DPPH radical-scavenging activity of the eluted fractions PSP-1, PSP-2 from RP-HPLC and GSH at the concentration of 250 μg/mL. All data were reported as mean ± SD (n = 3) and * indicated statistical difference (*p* < 0.05).

Perilla seed protein hydrolysate was separated into three fractions (a, b, c) with a Sephadex G-25 column (Fig 1A). Fraction c with the longest retention time and lowest molecular weight showed the highest DPPH radical-scavenging activity (87.7%) at a concentration of 0.5 mg/mL. This was in accordance with previous reports that peptides with smaller molecular weight from protein enzymatic hydrolysates had better antioxidant activity[6, 14]. Fraction c was further purified on a semipreparative C-18 column and separated into over forty portions based on molecular polarity (Fig 1C). Among them, two peaks (named PSP-1 and PSP-2) showed higher DPPH radical-scavenging activity (49.47% and 51.45%) at the concentration of 0.25 mg/mL (Fig 1D). Higher proportions of hydrophobic amino acids in peptides are correlated with better antioxidant activity, which is considered as the key determinant of the peptide’s ability to scavenge free radicals[15]. This could be due to the aromatic and sulfur containing chemistries of hydrophobic residues that make them sequester radicals.

### Identification of antioxidant peptides by LC-ESI-MS/MS

**Fig 2:**
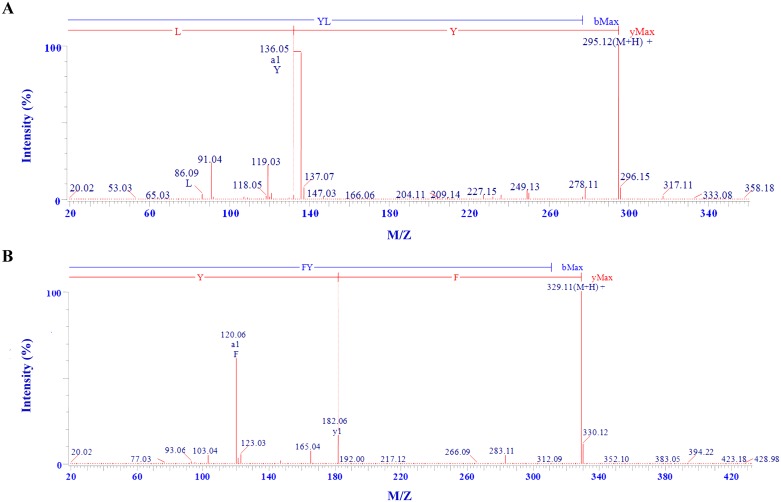
Identification of the molecular mass and amino acid sequences of PSP-1(A) and PSP-2(B) by Q-TOF MS/MS.

Analyzed by LC-ESI-MS/MS, the sequences of PSP-1 and PSP-2 were determined to be Tyr-Leu (YL) and Phe-Tyr (FY) with molecular weights (MWs) of 294.33 Da ([M+H]^+^ 295.12 Da) and 328.33 Da ([M+H]^+^ 329.11 Da), respectively (Fig 2). Many reported dipeptides isolated from plant proteins showed *in vitro* antioxidant effects [16]. In this study, the two isolated dipeptides each included a hydrophobic amino acid, Leu and Phe, respectively. Hydrophobic amino acids enhance peptide solubility in lipid and facilitate reactivity with hydrophobic radical species such as DPPH radicals [17]. According to our database search, FY is a novel peptide that has never been reported. On the other hand, although the sequence of YL has been reported, its purification process and antioxidant activity have not been previously explored[18]. Therefore, we performed comprehensive analyses on the activity of the two peptides.

### Antioxidant activity of synthesized peptides

#### ABTS radical-scavenging activity

**Fig 3:**
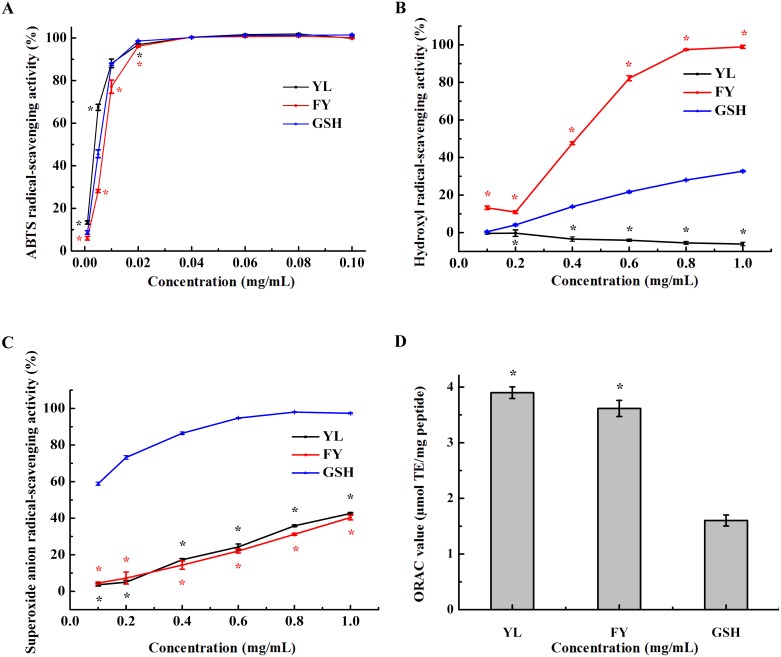
Free radical-scavenging activity assays of YL and FY. **A, B, C and D represent ABTS radical-scavenging activity, hydroxyl radical scavenging activity, superoxide anion radical-scavenging activity and ORAC assay, respectively**. The antioxidant peptide GSH was used as positive control. All data were reported as mean ± SD (n = 3) and * indicated statistical difference (*p* < 0.05).

ABTS radical-scavenging activity of YL and FY was examined at different concentrations. As shown in Fig 3A, YL and FY exhibited concentration-dependent activity. The ABTS radical-scavenging activity approached 100% when the peptide concentration was above 0.02 mg/mL. The IC_50_ values of YL, FY and GSH were 3.71 μg/mL, 7.23 μg/mL and 5.52 μg/mL, respectively. Obviously, YL and FY both showed 100% activity to quench ABTS radicals compared with the positive control (GSH) when the concentration was above 0.04 mg/mL.

#### Hydroxyl radical-scavenging activity

The inhibitory activity of hydroxyl radicals of YL and FY is shown in Fig 3B. FY showed stronger hydroxyl radical-scavenging activity (IC_50_ value is 0.41 mg/mL) than GSH (IC_50_ value is over 1.00 mg/mL), and also displayed a positive activity-concentration correlation. Since hydroxyl radicals can destroy biological macromolecules easily, such as proteins, carbohydrates, lipids, and nucleic acids, removing hydroxyl radicals is an effective protection of the body against several diseases[19]. Thus, FY can serve as a scavenger to reduce or eliminate damages induced by hydroxyl radicals in food and biological systems to improve human health[20]. In contrast, YL not only did not show hydroxyl radicals-scavenging activity, but instead protected hydroxyl radicals. Although both peptides YL and FY contained tyrosine, FY with Tyr at the C-terminus showed higher hydroxyl radical-scavenging activity, suggesting the position of Tyr or the special structure of FY might be important for the activity of the peptide. The results reinforced that antioxidant activity of the peptide relies on its composition and structure.

#### Superoxide anion radical-scavenging activity

The superoxide anion scavenging effects of YL and FY were investigated. Dose-dependent effects were observed containing the range of peptide concentrations from 0 to 1.0 mg/ml (Fig 3C). YL and FY showed similar superoxide anion radical-scavenging activity (42.63 ± 0.47% and 40.36 ± 1.28%, respectively, at the concentration of 1.0 mg/mL, *p* > 0.05). YL and FY had the ability to scavenge superoxide anion radicals, although weaker than that of GSH (97.36 ± 0.47% at 1.0 mg/mL, *p* < 0.05). The effect of scavenging superoxide anion radicals might work through interfering with the oxidant chain reaction.

#### ORAC assay

ORAC assay has been employed extensively in the measurement of antioxidant and oxidative stress[21]. The ORAC values of YL and FY were 3.90 ± 0.10 and 3.62 ± 0.14 μmol TE/mg peptide, respectively, higher than that of GSH (1.60 ± 0.10 μmol TE/mg peptide) (Fig 3D). Therefore, YL and FY possessed strong physiological radical-scavenging activity and might exert antioxidant effects in the human body[22].

### Inhibition of lipid peroxidation

**Fig 4:**
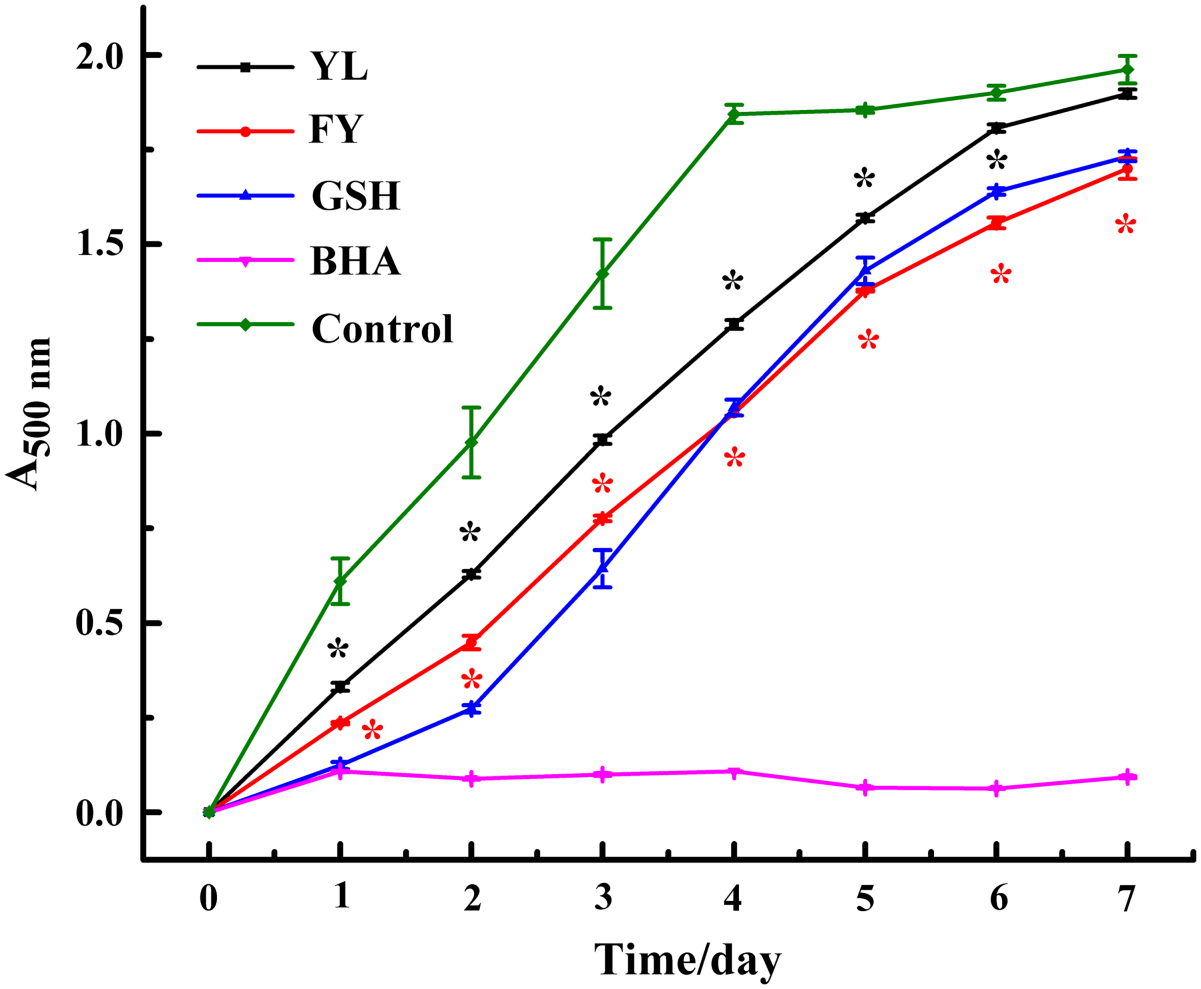
Linoleic acid peroxidation inhibition assay of YL and FY. The degree of lipid oxidation was measured at 24 h intervals. All data were reported as mean ± SD (n = 3) and * indicated statistical difference (*p* < 0.05).

Lipid peroxidation, developed with radical-mediated hydrogen atom abstraction from methylene carbons of polyunsaturated fatty acids, is thought to be an important damage mechanism of oils and fats [23]. YL and FY were compared with common antioxidants GSH and BHA with respect to linoleic acid peroxidation inhibition activity for 7 days. As shown in Fig 4, approximately 30% inhibition by YL and over 40% inhibition by FY were observed up to the fourth day compared with the blank. There was no significant difference in inhibition activity between FY and GSH after 4 days (*p* > 0.05). But YL and FY showed lower activity than BHA (*p* < 0.05). Both antioxidant dipeptide FY and YL possess hydrophobic amino acids Phe, Leu and Tyr. The presence of hydrophobic amino acids may increase the solubility of peptides in lipids, thereby facilitating interaction with radical species and leading to higher antioxidant activity[24]. The antioxidant peptides YL and FY could effectively reduce lipid peroxidation and extend the shelf life of oleaginous food.

### Inhibition of lipid peroxidation in the rat liver

**Fig 5:**
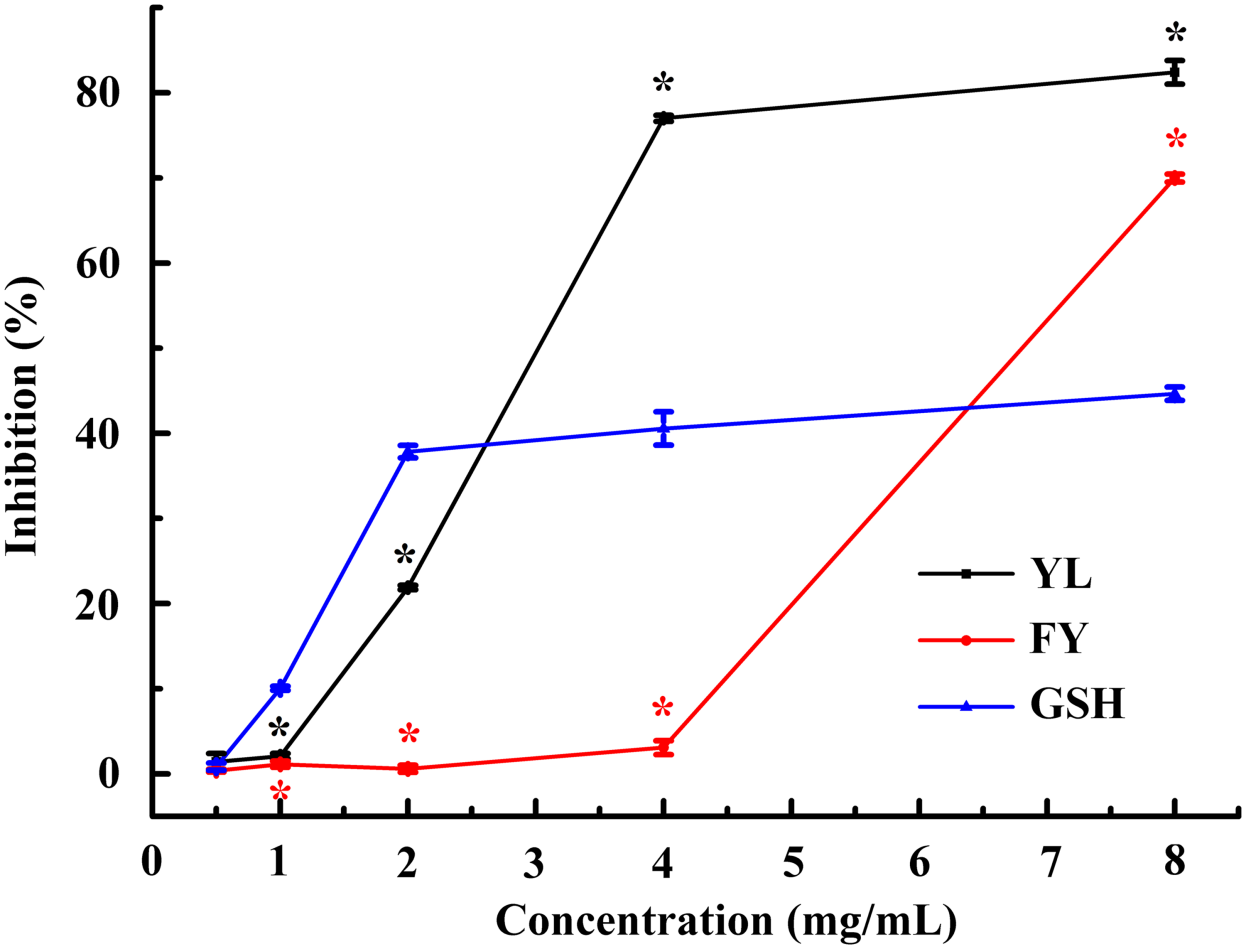
Inhibition of lipid peroxidation in rat liver by YL and FY. All data were reported as mean ± SD (n = 3) and * indicated statistical difference (*p* < 0.05).

As shown in Fig 5, YL and FY exhibited higher inhibition of lipid peroxidation in the rat liver (IC_50_ values are 3.02 mg/mL and 6.81 mg/mL, respectively) than the positive control GSH (IC_50_ value is over 8.00 mg/mL, *p* < 0.05). Similarly, YL also showed higher ORAC value than FY, implying that the similar radical scavenging mechanism might exert on the lipid peroxidation inhibition in the rat liver. Therefore, YL and FY might be developed as a hepatinica.

### Cytotoxicity effects on CHO cells and HepG-2 cells

**Fig 6:**
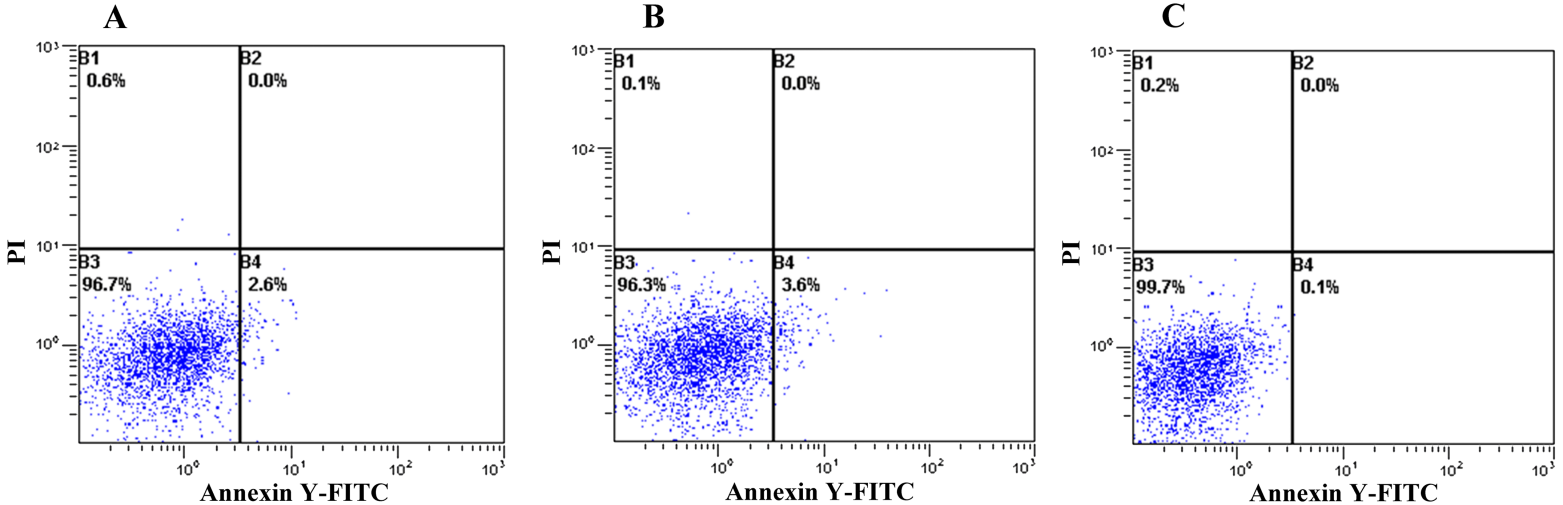
Cytotoxic effect of peptides YL and FY on CHO cells. A: control (un-treated CHO cells); B: CHO cells supplemented with 0.1 mg mL^-1^ YL; C: CHO cells supplemented with 0.1 mg mL^-1^ FY.

**Fig 7:**
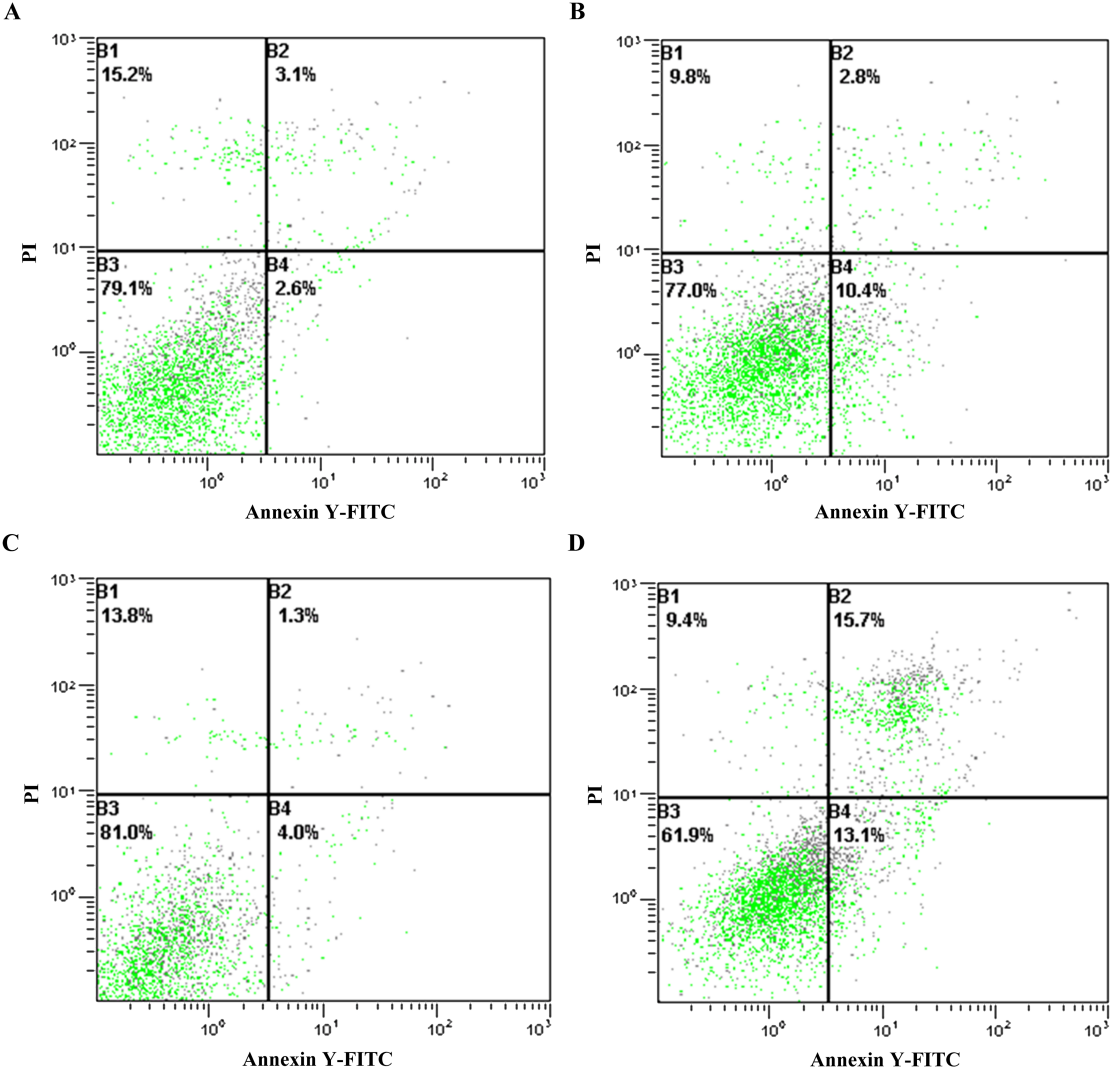
Cytotoxic effect of peptides YL and FY on HepG-2 cells. A: control (un-treated HepG-2 cells); B: HepG-2 cells supplemented with 0.1 mg mL^-1^ YL; C: HepG-2 cells supplemented with 0.05 mg mL^-1^ FY; D: HepG-2 cells supplemented with 0.1 mg mL^-1^ FY.

In order to detect cytotoxicity of YL and FY, high concentrations of YL and FY were added to the culture medium, and then CHO and HepG-2 cell viability was examined. After incubation with 0.1 mg/mL YL or FY, the cellular state of CHO cells did not change (Fig 6), indicating that 0.1mg/mL YL/FY did not have adverse effects on the proliferation of CHO cells. HepG-2 cells also remained unchanged after treatment with 0.1 mg/ml YL (Fig 7A and 7B). or 0.05 mg/mL FY (Fig 7A and 7C). However, 0.1 mg/mL FY can induce apoptosis in HepG-2 cells (Fig 7A and 7D). Therefore, the antioxidant dipeptide FY did not affect the proliferation of CHO cells but could induce HepG-2 cell apoptosis at a higher concentration (0.1 mg/mL). Thus, FY might act as an antioxidant in HepG2 cells and inhibit cancer cell proliferation, which was consistent with previous research showing that high antioxidant activity might effectively prevent cancer progression[25]. Nonetheless, the underlying anticancer mechanism of FY needs further investigation.

### Cytoprotection effects of YL and FY

**Fig 8:**
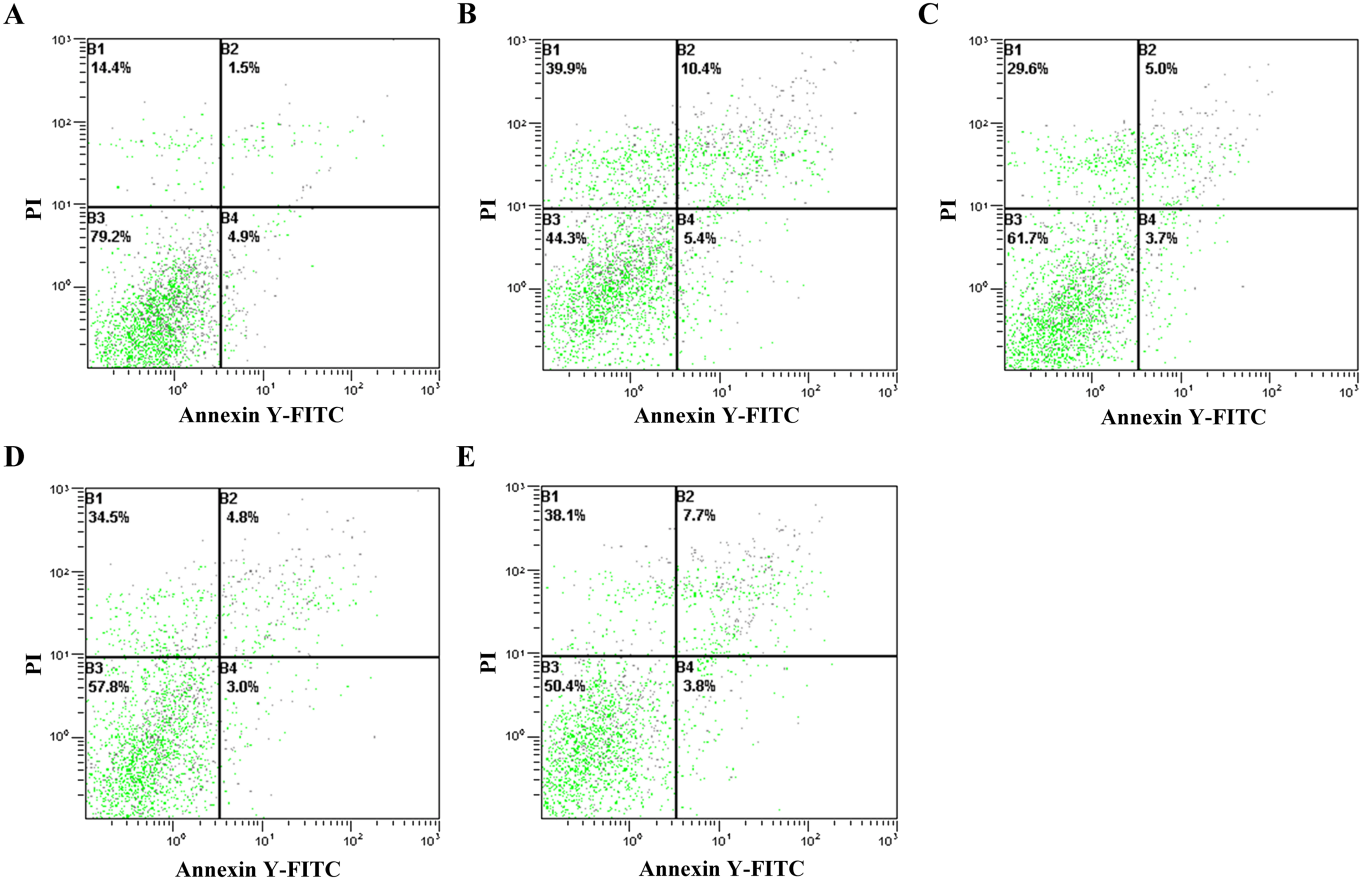
Cytoprotection effect of YL and FY on HepG-2 cells against H_2_O_2_-induced oxidative stress. A: control group (un-treated HepG-2 cell); B: H_2_O_2_ treatment group (HepG-2 cells +1 mmol L^-1^ H_2_O_2_); C: HepG-2 cells+1 mmol L^-1^ H_2_O_2_+0.1 mg mL^-1^ YL; D: HepG-2 cells+1 mmol L^-1^ H_2_O_2_+ 0.05 mg mL^-1^ FY; E: HepG-2 cells+1 mmol L^-1^ H_2_O_2_+ 0.1 mg mL^-1^ FY.

When HepG-2 cells were exposed to 1 mM hydrogen peroxide for 6 h, the percentage of normal cells reduced from 79.2% to 44.3% (*p* < 0.05), which was subsequently used to evaluate effects of the peptides on H_2_O_2_-induced oxidative damage (Fig 8A and 8B). Normal cell ratios were elevated to 61.7% and 57.8% (*p* < 0.05), respectively, when HepG-2 cells were pretreated by 0.1 mg/mL YL and 0.05 mg/mL FY for 18 h before H_2_O_2_ exposure (Fig 8C and 8D). Therefore, the antioxidant peptides YL and FY possessed protective effects against H_2_O_2_-induced oxidative stress in HepG-2 cells. Similar results have been reported from bioactive peptides derived from casein[26] and ovotransferrin hydrolysates [27]. Normal cell ratio was smaller when HepG-2 cells were pretreated with 0.1 mg/ml FY rather than that with 0.05 mg/mL FY, which was not surprising considering that 0.1 mg/mL FY could induce HepG-2 cell apoptosis. It is interesting that FY could protect against oxidative damage at the low concentration and induce apoptosis at the high concentration. Further research should be done to elaborate the antioxidant activity of FY in cancer cells.

### Structure-activity relationship of the antioxidant peptides

The antioxidant activity of bioactive peptides depends on the amino acid sequence and composition. Some di- and tri-peptides containing aromatic amino acid residues (Trp or Tyr), as well as His, show good antioxidant effects [28]. Tyr-residue is found in both YL and FY; Tyr can readily scavenge free radicals by donating a hydrogen atom from its phenolic group [29]. Hernandez-Ledesma *et al*. compared antioxidant activity of all amino acids by ORAC assay, and found that Trp showed the highest antioxidant activity, followed by Tyr, Met, Cys, His and Phe, whereas other amino acids did not exhibit antioxidant activity [30].

**Fig 9:**
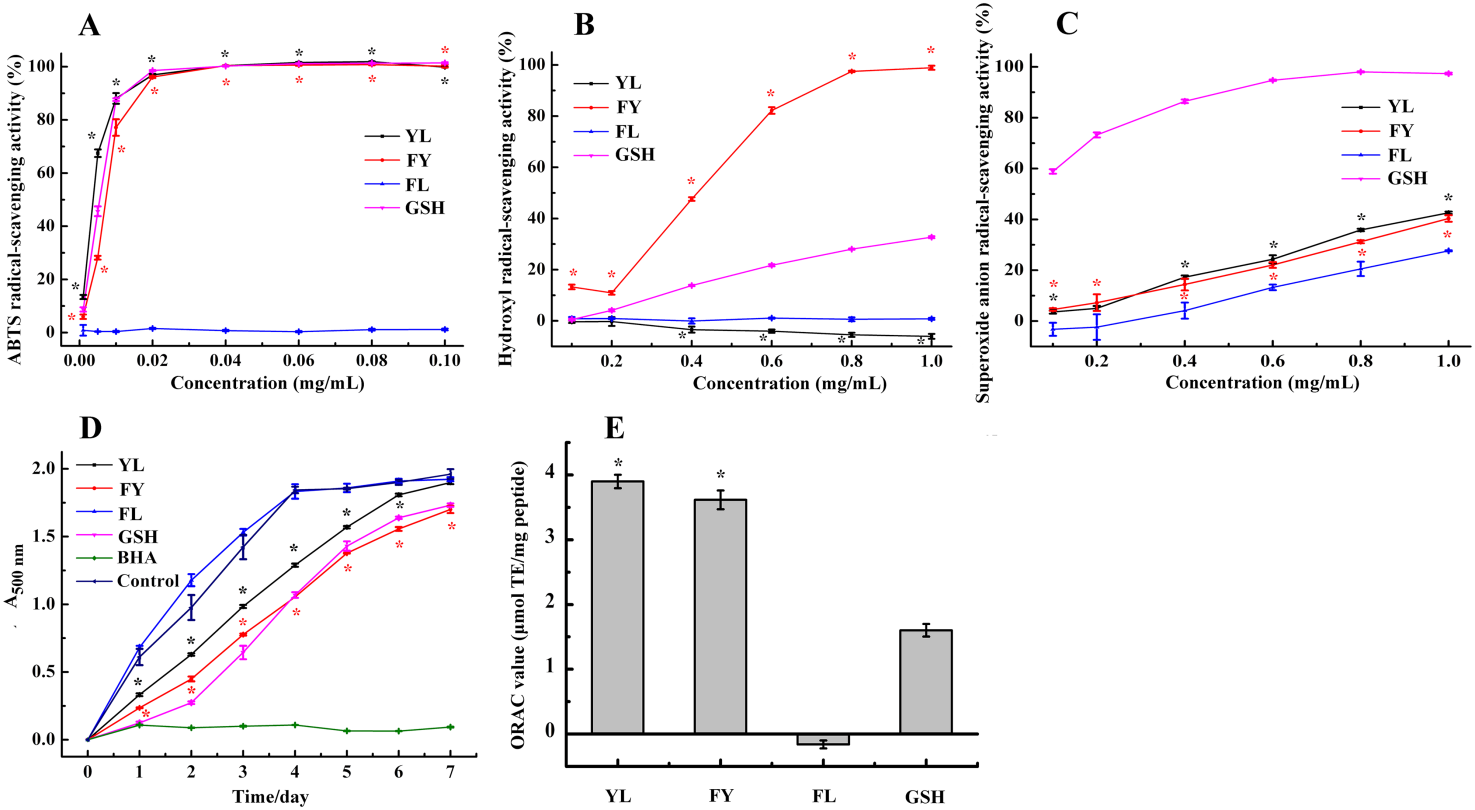
Comparison of antioxidant activity of the mutational peptide FL with the parent peptides YL and FY. A: ABTS radical-scavenging activity; B: hydroxyl radical-scavenging activity; C: superoxide anion radical-scavenging activity; D: linoleic acid peroxidation inhibition activity; E: ORAC activity. The antioxidant peptide GSH was used as positive control. All data were reported as mean ± SD (n = 3) and * indicated statistical difference between YL/FY and FL. (*p* < 0.05).

To confirm the crucial role of Tyr in the antioxidant activity of YL and FY, we designed a mutational dipeptide Phe-Leu (FL) and compared its antioxidant activity with that of YL and FY. The results are shown in Fig 9. Comparing with YL and FY, the ABTS radical-scavenging activity, oxygen radical absorbance capacity (ORAC) and linoleic acid peroxidation inhibition activity of FL was close to zero, demonstrating that the Tyr residue plays a vital role in bioactivity of both peptides. The superoxide anion radical-scavenging activity of FL was only half of YL and FY, indicating that Phe or Leu plays a key role besides Tyr-residue. The hydroxyl radical-scavenging activity of FL is close to that of YL, but much smaller than that of FY, suggesting that the C-terminal Tyr-residue is important for the hydroxyl radical-scavenging activity. The peptides with different sequence may adopt different structure and interact with radicals in different ways and show different activities. The mutational peptide FL still maintained some superoxide anion radical-scavenging activity, probably because of the presence of Phe, which has a benzene group to serve as the hydrogen donor [31].

## Conclusions

Two dipeptides with high antioxidant activity were purified from defatted perilla seed protein enzymatic hydrolysate. They were identified as YL and FY. The two peptides exhibited good radical scavenging activity and capacity of inhibiting lipid peroxidation in the rat liver. YL and FY also exerted strong cellular protection against oxidative stress. The Tyr residue was indispensable for the antioxidant activity of YL and FY. The results suggest that protein hydrolysates and purified peptides from perilla seeds have potential applications as food additives to ensure food quality, or as ingredients in medical and cosmetic industries to manage metabolic disorders that arise from excessive levels of ROS. However, *in vivo* antioxidant activity of the two peptides and the mechanism underlying their protection of cellular oxidative stress protection need further research. In the meantime, apoptosis-induction of HepG2 cells by the high concentration of FY should be further investigated.
